# Draft Genome Sequence of Oleaginous Yeast *Saitozyma* sp. Strain JCM 24511, Isolated from Soil on Iriomote Island, Okinawa, Japan

**DOI:** 10.1128/MRA.00196-20

**Published:** 2020-11-25

**Authors:** Masako Takashima, Ri-ichiroh Manabe, Moriya Ohkuma

**Affiliations:** a Japan Collection of Microorganisms, RIKEN BioResource Research Center, Tsukuba, Ibaraki, Japan; b Division of Genomic Technologies, RIKEN Center for Life Science Technologies, Yokohama, Kanagawa, Japan; c Laboratory for Comprehensive Genomic Analysis, RIKEN Center for Integrative Medical Science, Yokohama, Kanagawa, Japan; University of California, Riverside

## Abstract

Here, we report draft genome sequence of oleaginous yeast strain *Saitozyma* sp. JCM 24511, which is phylogenetically closely related to Saitozyma podzolica. These data will have implications not only for the study of the oleaginous activities of yeasts but also for the study of the plant-microorganism microbiome.

## Abstract (precis)

Here, we report draft genome sequences of oleaginous yeast strain *Saitozyma* sp. JCM 24511, which is phylogenetically closely related to Saitozyma podzolica. These data will have implications not only for the study of the oleaginous activities of yeasts but also for the study of the plant-microorganism microbiome.

## ANNOUNCEMENT

*Saitozyma* sp. strain JCM 24511 was isolated from soil collected on Iriomote Island in Okinawa (southern part of Japan; 24°17′N, 123°51′E; 289.61 km^2^) (1) and was found to be phylogenetically closely related to Saitozyma podzolica (Tremellales, Agaricomycotina, Basidiomycota). Tanimura et al. (2) reported that it is an oleaginous yeast with high lipid productivity. Genome sequences of oleaginous yeasts have been reported (e.g., Rhodosporidium toruloides [present name Rhodotorula toruloides] [3]). Regarding the *Saitozyma* species, the draft genome sequence of *Saitozyma podzolica* (formerly Cryptococcus podzolicus) DSM 27192, isolated in Germany, is available (4). *Saitozyma podzolica,* whose type strain was isolated from podzolic soil in Siberia, Russia, has been frequently isolated from soil, and its biotechnological importance has been reviewed (5, 6). *Saitozyma* sp. JCM 24511 would be a separate oleaginous species from *S. podzolica* based on the sequence identity of the D1/D2 domain of the 26S rRNA gene (99.3%, 539/543 nucleotides) according to the guideline by Vu et al. (7).

To contribute to the understanding of the roles that *Saitozyma* species play in the environment, we determined the draft genome sequence for *Saitozyma* sp. JCM 24511. Cells grown on YM agar (BD-Difco) plates at 25°C were harvested after 3 days of cultivation. The genomic DNA was prepared from freeze-dried cell pellets according to the method of Raeder and Broda (8) and purified using a Genomic-tip 100/G (Qiagen, Tokyo, Japan) following the manufacturer’s instructions.

Two libraries, having approximate insert sizes of 240 bp and 3 kbp, were prepared from DNA using a TruSeq DNA PCR-free library preparation kit and a Nextera mate pair sample preparation kit with some modifications (9). Genome sequencing was performed using an Illumina HiSeq 2500 instrument with 151-bp paired-end reads. The acquired reads were assembled using ALLPATHS-LG (v.52488) (10). Protein-coding genes were predicted using the MAKER annotation pipeline (2.31.8) in collaboration with AUGUSTUS (3.0.3) and SNAP (2013-02-16), of which both were trained with the Cryptococcus neoformans var. *neoformans* JEC21 sequence (GenBank assembly accession no. GCA_000091045.1), as well as GeneMark-ES (4.21) (11). The genes were annotated using Sma3s (12) based on the Uniprot-TrEMBL and UniProt Swiss-Prot databases (release 2015_11). tRNA, small noncoding RNAs, and transposons were also annotated using tRNAscan-SE (1.23) (13), Infernal cmscan (1.1.1) (14), and the Rfam database (release 12.0) (15), as well as RepeatRunner (16) and RepeatMasker (open-4.0.5) (http://www.repeatmasker.org/) based on Dfam v.2.0 (17). Assembly and annotation are summarized in Table 1.

**TABLE 1:** Summary of assembly and annotation of genomic data of *Saitozyma* sp. JCM 24511

Parameter	Value
Sum read length (Mbp)	2,030
No. of scaffolds	55
No. of contigs	234
Total length (Mbp)	28.2
Coverage (×) (gaps [%])	72 (1.1)
Scaffold *N*_50_ (kb)	1,282
GC content (%)	58.5
CEGMA^*a*^ score (%)	93.2
No. of protein-coding genes	10,192
No. of functionally annotated genes by UniProt-TrEMBL	7,333
No. of functionally annotated genes by UniProt Swiss-Prot	2,072
Avg no. of exons per gene model	6.0
Avg exon size (bp)	247
Avg intron size (bp)	96
No. of tRNAs	57
No. of small noncoding RNAs	259
No. of transposons	448

a CEGMA, Core Eukaryotic Genes Mapping Approach.

Using next-generation sequencing, Toju et al. recently reported that *Saitozyma/Cryptococcus* species were important taxa in the metacommunity-scale network (18). Thus, we believe our genomic information will also have implications for the plant-microorganism microbiome.

### Data availability.

The genome sequence has been deposited at DDBJ/EMBL/GenBank under BioProject PRJDB3825, accession no. BCLC01000000, and SRA accession no. DRR043260 and DRR042694. The annotations are available via the homepage of the Japan Collection of Microorganisms (JCM) at the RIKEN BioResource Research Center (http://www.jcm.riken.jp/cgi-bin/nbrp/nbrp_list.cgi).
